# Geographic Distribution of Hantaviruses Associated with Neotomine and Sigmodontine Rodents, Mexico

**DOI:** 10.3201/eid1804.111028

**Published:** 2012-04

**Authors:** Mary L. Milazzo, Maria N.B. Cajimat, Hannah E. Romo, Jose G. Estrada-Franco, L. Ignacio Iñiguez-Dávalos, Robert D. Bradley, Charles F. Fulhorst

**Affiliations:** University of Texas Medical Branch, Galveston, Texas, USA (M.L. Milazzo, M.N.B. Cajimat, H.E. Romo, J.G. Estrada-Franco, C.F. Fulhorst);; Universidad de Guadalajara, Autlán de Navarro, Mexico (L.I. Iñiguez-Dávalos);; Texas Tech University, Lubbock, Texas, USA (R.D. Bradley)

**Keywords:** hantavirus, hantavirus pulmonary syndrome, *Bunyaviridae*, Mexico, rodents, viruses

## Abstract

To increase our knowledge of the geographic distribution of hantaviruses associated with neotomine or sigmodontine rodents in Mexico, we tested 876 cricetid rodents captured in 18 Mexican states (representing at least 44 species in the subfamily Neotominae and 10 species in the subfamily Sigmodontinae) for anti-hantavirus IgG. We found antibodies against hantavirus in 35 (4.0%) rodents. Nucleotide sequence data from 5 antibody-positive rodents indicated that Sin Nombre virus (the major cause of hantavirus pulmonary syndrome [HPS] in the United States) is enzootic in the Mexican states of Nuevo León, San Luis Potosí, Tamaulipas, and Veracruz. However, HPS has not been reported from these states, which suggests that in northeastern Mexico, HPS has been confused with other rapidly progressive, life-threatening respiratory diseases. Analyses of nucleotide sequence data from 19 other antibody-positive rodents indicated that El Moro Canyon virus and Limestone Canyon virus are geographically widely distributed in Mexico.

## Abstract 2

El Moro Canyon virus and Limestone Canyon virus are widely distributed and may cause hantavirus pulmonary syndrome.

Hantavirus pulmonary syndrome (HPS) is a potentially fatal zoonosis caused by hantaviruses (family *Bunyaviridae*, genus *Hantavirus*) that are principally associated with members of the rodent family Cricetidae, more specifically, members of the subfamily Neotominae or Sigmodontinae (*1**,**2*). The viruses known to cause HPS on the North American continent are Bayou virus, Black Creek Canal virus (BCCV), Choclo virus (CHOV), New York virus, and Sin Nombre virus (SNV) (*3**–**7*). Other hantaviruses that are principally associated with neotomine or North American sigmodontine rodents include Carrizal virus (CARV), Catacamas virus, El Moro Canyon virus (ELMCV), Huitzilac virus (HUIV), Limestone Canyon virus (LSCV), Montano virus (MTNV), Muleshoe virus (MULV), Playa de Oro virus, and Rio Segundo virus (RIOSV) (*8**–**14*).

Specific rodents (usually 1 or 2 closely related species) are the principal hosts of the hantaviruses, for which natural host relationships have been well characterized. The current principal host relationships of some hantaviruses seem to represent a long-term association between viruses in the genus *Hantavirus* and rodents in the family Cricetidae. Evidence for this ancient relationship includes the association of phylogenetically closely related hantavirus species with phylogenetically closely related allopatric rodent species. For example, Catacamas virus is associated with Coues’s rice rat (*Oryzomys couesi*) in Honduras, and Bayou virus is associated with the marsh rice rat (*Oryzomys palustris*) in the southeastern United States (*9**,**15*).

The rodent fauna of Mexico comprises the brush mouse (*Peromyscus boylii*), the deer mouse (*P. maniculatus*), the western harvest mouse (*Reithrodontomys megalotis),* the hispid cotton rat (*Sigmodon hispidus*), the fulvous pygmy rice rat (*Oligoryzomys fulvescens*), and 122 other species in the Neotominae or Sigmodontinae (*16*). In the southwestern United States, LSCV, SNV, ELMCV, and MULV are principally associated with rodents of the species *P. boylii*, *P. maniculatus*, *R. megalotis,* and *S. hispidus*, respectively (*10**–**12**,**17*), and that in Panama, CHOV is principally associated with *O. fulvescens* (*18*). Hypothetically, LSCV, SNV, ELMCV, and/or MULV—in association with deer mice (*Peromyscus* spp.), harvest mice (*Reithrodontomys* spp.), or cotton rats (*Sigmodon* spp.)—are widely distributed in northern Mexico, and the hantavirus assemblage of southern Mexico includes CHOV or hantaviruses that are phylogenetically closely related to CHOV.

Our knowledge of the rodent-associated hantaviruses in Mexico includes the following findings: HUIV RNA in a western harvest mouse (*R. megalotis*) captured in Morelos (*8*); CARV RNA in a Sumichrast’s harvest mouse (*R. sumichrasti*) and MTNV RNA in an Orizaba deer mouse (*P. beatae*) from Guerrero (*8*); Playa de Oro virus RNA in a Mexican oryzomys (*Oryzomys mexicanus*) and Jaliscan cotton rat (*S. mascotensis*) from Colima (*13*); ELMCV RNA and SNV RNA in western harvest mice from Zacatecas (*14*); antibody against hantavirus in nimble-footed mice (*P. levipes*) captured in Tamaulipas (*19*); and antibody against hantavirus in a North American deer mouse (*P. maniculatus*), transvolcanic mice (*P. hylocetes*), black-eared mice (*P. melanotis*), and Sumichrast’s harvest mouse captured in the state of Mexico (*20**,**21*). The purpose of this study was to extend our knowledge of the geographic distribution of hantaviruses associated with neotomine or sigmodontine rodents in Mexico.

## Materials and Methods

Blood samples from 876 rodents, representing at least 44 species in the Neotominae and 10 species in the Sigmodontinae, were tested for anti-hantavirus IgG. The 876 rodents were captured during 1998–2008 at 43 localities in 18 states in Mexico (Table A1). Blood samples from all of the rodents and lung samples from the antibody-positive rodents were acquired from the Natural Science Research Laboratory, Museum of Texas Tech University, Lubbock, Texas, USA.

The blood samples were tested for IgG to Caño Delgadito virus (CADV) strain VHV-574 by using an ELISA in which CADV can be highly cross-reactive with SNV, BCCV, and other North American hantaviruses (*22*). The antibody titers in the antibody-positive blood samples were recorded as 320, 1,280, or >5,120.

Samples of lung tissue from the antibody-positive rodents were tested for hantavirus nucleocapsid (N) protein gene RNA. Subsequently, we determined the nucleotide sequences of a 1,078-nt fragment of the glycoprotein precursor (GPC) genes of 11 of the hantaviruses associated with the antibody-positive rodents. We chose these 11 viruses to represent the geographic distribution and natural host associations of the hantaviruses in Mexico included in this study. Total RNA was isolated from 30 mg to 45 mg of lung tissue by using Tri Reagent (Sigma-Aldrich, St. Louis, MO, USA). First-strand cDNA was synthesized from small (S) segment and medium (M) segment RNA by using SuperScript II RNase H^–^ Reverse Transcriptase (Invitrogen Life Technologies, Inc., Carlsbad, CA, USA) in conjunction with oligonucleotide 5′-GGTGGTTGTGGTAGTAGTAGACTCC-3′ (*23*). The first-round and second-round (hemi-nested) PCR assays used the MasterTaq Kit (Eppendorf North America, Inc., Westbury, NY, USA). (The sequences of the oligonucleotides that were used to prime the PCR are available from the corresponding author.) The sizes of the N protein gene amplicons from the second-round assays ranged from 377 to 545 bp, the sizes of the GPC gene amplicons from the second-round assays ranged from 607 to 631 bp (M1 amplicon) and 571 to 618 bp (M2 amplicon), and the lengths of the overlaps between the M1 and M2 amplicons ranged from 125 to 134 bp. Together, the nucleotide sequences of the M1 and M2 amplicons encoded a 359-aa fragment of the G_C_ glycoprotein.

The sequences in each dataset were aligned by using the computer program ClustalW version 2.0.12 (*24*). Sequence nonidentities were equivalent to uncorrected (p) distances. The phylogenetic analyses of nucleotide sequences were conducted with MRBAYES 3.1.2 (*25*) and programs in the computer software package PAUP* (*26*). The Bayesian analyses used the general time reversible + proportion invariant + Γ model and the following options in MRBAYES 3.1.2: two simultaneous runs of 4 Markov chains, 2 million generations, and sample frequency = every 1,000th generation. The first 1,000 trees were discarded after review of the likelihood scores, convergence statistics, and potential scale reduction factors; and a consensus tree (50% majority rule) was constructed from the remaining trees. Probability values in support of the clades were calculated a posteriori, and clades with probability values >0.95 were considered supported by the data (*27*).

## Results

**Figure 1:**
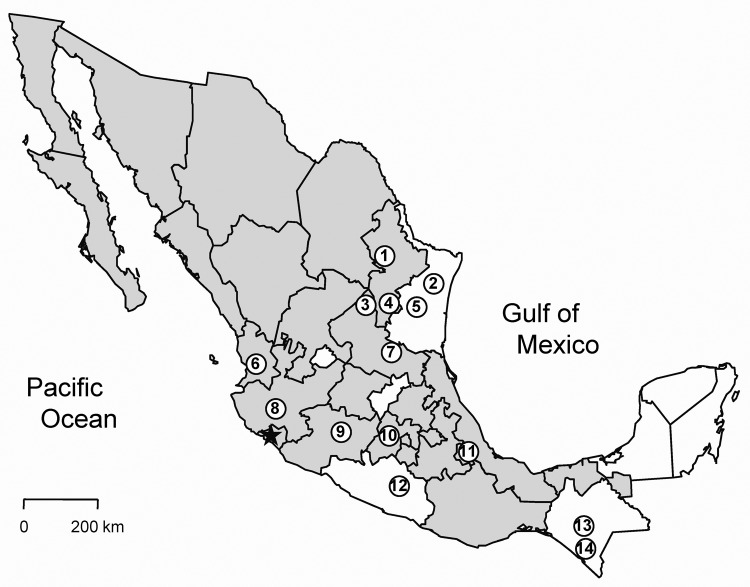
Municipalities in Mexico in which rodents positive for antibodies against hantaviruses were captured: 1) Municipality of Santiago, state of Nuevo León; 2) San Fernando, Tamaulipas; 3) Real de Catorce, San Luis Potosí; 4) Doctor Arroyo, Nuevo León; 5) Soto la Marina, Tamaulipas; 6) Santa María del Oro, Nayarit; 7) Ciudad del Maíz, San Luis Potosí; 8) Autlán de Navarro, Jalisco; 9) Uruapan, Michoacán; 10) Ecatepec de Morelos, México; 11) Perote, Veracruz; 12) Chilpancingo de los Bravo, Guerrero; 13) Ocozocoautla de Espinosa, Chiapas; 14) Mapastepec, Chiapas. The states shaded in gray are those in which members of *Peromyscus maniculatus* have been found (*28*). The star indicates the location in Colima at which rodents infected with Playa de Oro virus were captured in a previous study (*13*).

Antibody (IgG) against hantavirus was found in 35 (4.0%) of 876 rodents captured in 18 states in Mexico (Table A1). The antibody-positive rodents were from 16 localities (Table A2) in 14 municipalities in 10 states: Chiapas, Guerrero, Jalisco, México, Michoacán, Nayarit, Nuevo León, San Luis Potosí, Tamaulipas, and Veracruz (Figure 1). None of the rodents captured in Chihuahua (n = 9), Coahuila (n = 16), Guanajuato (n = 8), Oaxaca (n = 64), Puebla (n = 15), Sinaloa (n = 9), Sonora (n = 22), or Tlaxcala (n = 16) were antibody-positive to CADV strain VHV-574.

**Figure 2:**
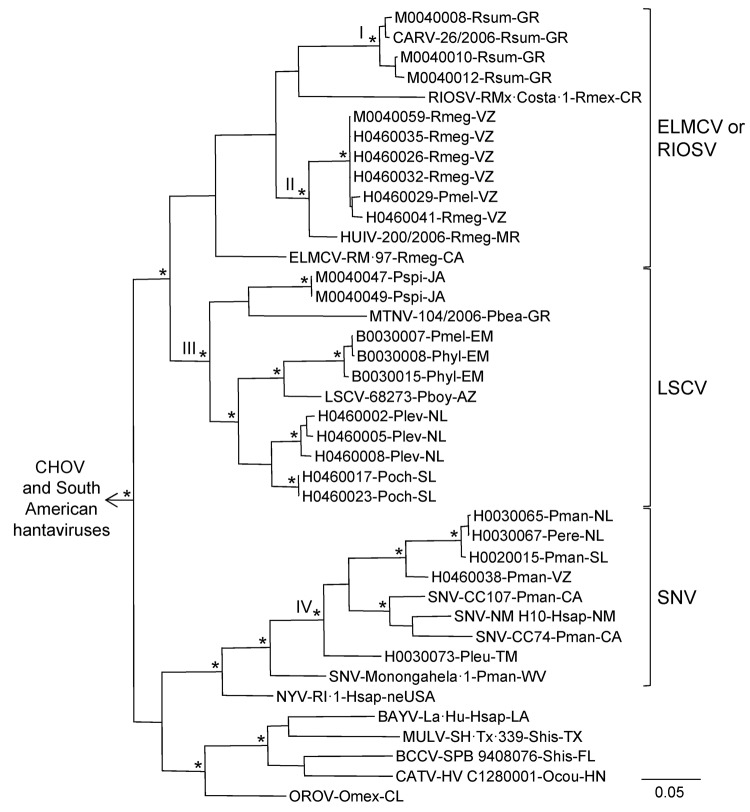
Results of the Bayesian analyses of the nucleotide sequences of a fragment of the nucleocapsid protein genes of the 24 hantaviruses found in Mexico in this study and 22 other hantaviruses naturally associated with members of the Neotominae or Sigmodontinae. An asterisk at a node indicates that the probability values in support of the clade were >0.95. Scale bar indicates substitutions per site. The Roman numerals indicate the phylogenetic groups represented by the hantaviruses from Mexico in this study. The branch labels include (in the following order) virus, strain, host species, and state or country. BAYV, Bayou virus, strain LA-Hu (GenBank accession no. L36929); BCCV, Black Creek Canal virus, SPB 9408076 (L39949); CARV, Carrizal virus, 26/2006 (AB620103); CATV, Catacamas virus, HV C1280001 (DQ256126); CHOV, Choclo virus, 588 (DQ285046); ELMCV, El Moro Canyon virus, RM-97 (U11427); HUIV, Huitzilac virus, 200/2006 (AB620106); LSCV, Limestone Canyon virus, 68273 (AF307322); MTNV, Montano virus, 104/2006 (AB620100); MULV, Muleshoe virus, SH-Tx-339 (U54575); NYV, New York virus, RI-1 (U09488); OROV, Playa de Oro virus (EF534079); RIOSV, Rio Segundo virus, RMx-Costa-1 (U18100); and SNV, Sin Nombre virus strains Convict Creek 74 (CC74), Convict Creek 107 (CC107), Monongahela-1, and NM H10 (L33816, L33683, U32591, and L25784, respectively). The viruses found in South America were Andes virus, strain Chile-9717869 (GenBank accession no. AF291702); Caño Delgadito virus, VHV-574 (DQ285566); Laguna Negra virus, 510B (AF005727); Maporal virus, HV 97021050 (AY267347); and Rio Mamoré virus, HTN-007 (FJ532244). Locations: AZ, Arizona; CA, California; CL, Colima; CR, Costa Rica; EM, México (state); FL, Florida; GR, Guerrero; HN, Honduras; JA, Jalisco; LA, Louisiana; MR, Morelos; NL, Nuevo León; NM, New Mexico; neUSA, northeastern United States (New York or Rhode Island); SL, San Luis Potosí; TM, Tamaulipas; TX, Texas; VZ, Veracruz; WV, West Virginia. Species: Hsap, *Homo sapiens*; Pbea, *Peromyscus beatae*; Pboy, *P. boylii*; Pere, *P. eremicus*; Phyl, *P. hylocetes*; Pleu, *P. leucopus*; Plev, *P. levipes*; Pman, *P. maniculatus*; Pmel, *P. melanotis*; Poch, *P. ochraventer*; Pspi, *P. spicilegus*; Ocou, *Oryzomys couesi*; Omex, *O. mexicanus*; Rmeg, *Reithrodontomys megalotis*; Rmex, *R. mexicanus*; Rsum, *R. sumichrasti*; Shis, *Sigmodon hispidus.* The designated outgroup was Andes virus strain Chile-9717869.

Hantavirus N protein gene RNA was detected in samples of lung from 24 (68.6%) of the 35 antibody-positive rodents (Table). The Bayesian analyses of the N protein gene sequences separated the 24 Mexican viruses in this study into 4 groups (Figure 2). Group I included CARV, group 2 included HUIV, group III included LSCV and MTNV, and group IV included SNV strains Convict Creek 74, Convict Creek 107, and NM H10.

**Figure 3:**
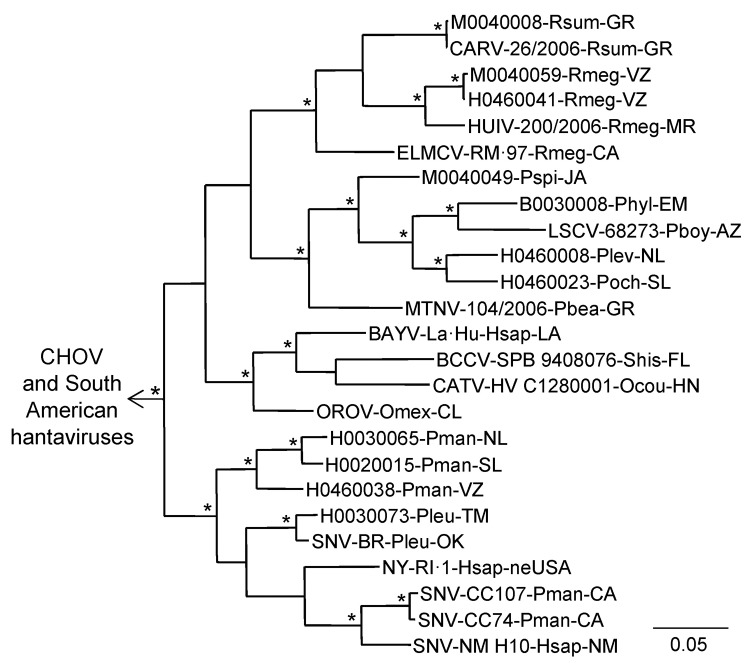
Results of the Bayesian analyses of the nucleotide sequences of a 1,078-nt fragment of the glycoprotein precursor genes of 11 of the 24 hantaviruses found in Mexico in this study and 20 other hantaviruses naturally associated with members of the Neotominae or Sigmodontinae. An asterisk at a node indicates that the probability values in support of the clade were >0.95. Scale bar indicates substitutions per site. The branch labels include (in the following order) virus, strain, host species, and state or country. BAYV, GenBank accession no. L36930; BCCV, L39950; CARV, AB620104; CATV, DQ177347; CHOV, DQ285047; ELMCV, U26828; HUIV, AB620107; LSCV, AF307323; MTNV, AB620101; NYV, U36801; OROV, EF534080; SNV—strains BR (AF030552), CC74 (L33684), CC107 (L33474), and NM H10 (L25783). The viruses from South America were Andes virus, GenBank accession no. AF291703; Caño Delgadito virus, DQ284451; Laguna Negra virus, AF005728; Maporal virus, AY363179; and Rio Mamoré virus, FJ608550. The designated outgroup was Andes virus strain Chile-9717869.

Hantavirus GPC gene RNA was detected in each of the 11 rodents assayed for GPC gene RNA (Table). The topology of the GPC gene tree (Figure 3) was essentially identical to the topology of the N protein gene tree (Figure 2) with respect to relationships between the viruses from Mexico in this study, CARV, HUIV, MTNV, and the other hantaviruses found in North America. M0040008, CARV, M0040059, H0460041, HUIV, and ELMCV were monophyletic in the Bayesian analyses of the GPC gene sequence data (Figure 3) and N protein gene sequence data (Figure 2).

Nonidentities among the amino acid sequences of the 359-aa fragment of the G_C_ glycoproteins of the 11 hantaviruses from Mexico in this study, CARV, HUIV, and MTNV ranged from 0% to 18.4% (Table A3). Nonidentities between the sequences of the 359-aa fragment of the G_C_ glycoproteins of these 14 hantaviruses and the sequences of the homologous fragment of the G_C_ glycoproteins of the other hantaviruses found in North America ranged from 1.1% (H0030073 and SNV strain Blue River-Oklahoma) to 18.9% (M0040049 and BCCV strain SPB 9408076).

## Discussion

The Eighth Report of the International Committee on Taxonomy of Viruses sets forth the criteria for species demarcation in the genus *Hantavirus* (*1*). One of these criteria is that strains of different species must exhibit at least a 7% difference in amino acid sequence identity in comparisons of complete N protein sequences and in comparisons of complete GPC sequences.

ELMCV was first described in 1994 (*10*); LSCV was described in 2001 (*11*); and CARV, HUIV, and MTNV were described in 2011 (*8*). In a previous study (*8*), the amino acid sequence of the N protein of MTNV was 5.8% different from the amino acid sequence of the N protein of LSCV strain 68273, and the amino acid sequence of the N protein of CARV was 3.7% different from the amino acid sequence of the N protein of ELMCV strain RM•97 and 8.4% different from the amino acid sequence of the N protein of RIOSV strain RMx•Costa•1. The amino acid sequence of the N protein of HUIV was 1.4% different from the amino acid sequence of the N protein of ELMCV strain RM•97 and 8.4% different from the amino acid sequence of the N protein of RIOSV strain RMx•Costa•1. Accordingly, MTNV should be considered a strain of LSCV, and CARV and HUIV could be considered strains of ELMCV or RIOSV. Alternatively, CARV and HUIV could be considered members of a species complex that includes ELMCV, RIOSV, and other hantaviruses that are naturally associated with harvest mice (*Reithrodontomys* spp.). There is presumptive evidence for RIOSV or hantavirus(es) that are genetically closely related to RIOSV in Sumichrast’s harvest mice, a Mexican harvest mouse (*R. mexicanus*), and a Chiriqui harvest mouse (*R. creper*) captured in Panama (*29*).

Collectively, the results of the Bayesian analyses of N protein gene sequence data (Figure 2), Bayesian analyses of the GPC gene sequence data (Figure 3), and pairwise comparisons of G_C_ sequences (Table A3) indicate that H0020015, H0030065, H0030073, and H0460038 are strains of SNV. The results of these analyses also indicate that M0040008, M0040059, and H0460041 are strains of ELMCV or RIOSV and that B0030008, H0460008, H0460023, and M0040049 are strains of LSCV. Accordingly, the N protein gene RNA–positive rodents in this study (Table) were infected with SNV, ELMCV, RIOSV, or LSCV.

Specific knowledge of the natural host relationships of LSCV previously was limited to LSCV RNA in 6 brush mice (*P. boylii*) captured in northern Arizona (*11*). The results of this study indicate that the transvolcanic mouse (*P. hylocetes*) and the black-eared mouse (*P. melanotis*) in the state of México, the nimble-footed mouse (*P. levipes*) in Nuevo León, the El Carrizo deer mouse (*P. ochraventer*) in San Luis Potosí, and the gleaning mouse (*P. spicilegus*) in Jalisco are natural but not necessarily principal hosts of LSCV.

HPS was first recognized as a distinct clinical entity in the southwestern United States in 1993 (*30*). Through 2009, a total of 510 HPS cases were reported to the National Notifiable Diseases Surveillance System or registered by the Centers for Disease Control and Prevention (*31*). Most of these cases occurred in the southwestern United States, 92 (33.7%) of 273 HPS cases that occurred in the southwestern United States before 2010 were fatal, and all of the cases from the southwestern United States through 2009 were attributed to SNV.

The results of this study indicate that SNV is widely distributed in northeastern Mexico. The geographic distribution of deer mice (*P. maniculatus*) in Mexico includes 23 states (*28*), and ≈20 million persons lived in rural areas in this 23-state region in 2010 (*32*). Yet, to our knowledge, no cases of HPS have been reported from northeastern Mexico or elsewhere in Mexico.

We hypothesize that HPS caused by SNV in Mexico has been confused with other rapidly progressive, life-threatening respiratory diseases (e.g., plague, tularemia, pneumococcal pneumonia, influenza). Alternatively, SNV in Mexico is substantially less virulent than SNV in the western United States, or human contact with SNV-infected rodents in Mexico is less frequent or less intimate than human contact with SNV-infected rodents in the western United States.

Laboratory confirmation of the diagnoses of most HPS cases in the United States before 2010 was based on the results of serologic assays in which ELMCV and LSCV can be highly cross-reactive with SNV (*31*). Thus, in all likelihood, some of the HPS cases from the western United States were actually caused by ELMCV or LSCV, and these viruses as well as SNV are etiologic agents of HPS in Mexico.

## Appendix

**Table A1:** Rodents captured in Mexico and tested for antibody against hantavirus, 1998–2008, by species and state*

Species	No. rodents (state)	Antibody prevalence†
*Baiomys musculus*	24 (CH), 3 (JA), 1 (MH), 14 (OA), 1 (VZ)	1/43
*B. taylori*	2 (CI), 1 (GJ), 1 (GR), 24 (JA), 5 (MH), 4 (PU), 9 (SI), 2 (TM)	1/48
*Habromys ixtlani*	5 (OA)	0/5
*Habromys* sp.	1 (VZ)	0/1
*Hodomys alleni*	1 (OA)	0/1
*Megadontomys thomasi*	1 (GR)	0/1
*Neotoma albigula*	6 (SO)	0/6
*Neotoma leucodon*	13 (NL), 10 (SL)	0/23
*Neotoma mexicana*	1 (CH), 1 (MH), 3 (NA), 3 (OA), 2 (VZ)	0/10
*Neotoma micropus*	2 (TM)	0/2
*Neotoma picta*	2 (GR)	0/2
*Neotomodon alstoni*	6 (EM), 1 (MH)	0/7
*Oligoryzomys fulvescens*	1 (SL)	0/1
*Onychomys leucogaster*	1 (NL), 3 (SL), 1 (TM)	0/5
*Onychomys torridus*	3 (SO)	0/3
*Oryzomys alfaroi*	1 (OA)	0/1
*Oryzomys couesi*	7 (CH), 1 (OA), 2 (VZ)	1/10
*Oryzomys melanotis*	4 (SL)	0/4
*Oryzomys mexicanus*	3 (CH)	0/3
*Osgoodomys banderanus*	2 (JA), 11 (MH), 2 (NA)	0/15
*Peromyscus aztecus*	3 (CH), 1 (OA)	0/4
*P. beatae*	3 (CH), 6 (GR), 21 (OA), 4 (VZ)	0/34
*P. boylii*	3 (CI), 2 (JA), 6 (SO)	0/11
*P. difficilis*	1 (OA), 8 (TL)	0/9
*P. eremicus*	8 (CU), 5 (NL), 6 (SL)	1/19
*P. evides*	4 (GR)	0/4
*P. gratus*	5 (JA), 5 (PU)	0/10
*P. hooperi*	6 (CU)	0/6
*P. hylocetes*	8 (EM)	2/8
*P. leucopus*	1 (NL), 1 (SL), 13 (TM)	2/15
*P. levipes*	3 (EM), 2 (GR), 30 (NL), 16 (SL)	4/51
*P. maniculatus*	9 (NL), 5 (SL), 1 (TL), 5 (VZ)	3/20
*P. megalops*	29 (GR)	1/29
*P. melanophrys*	5 (GJ), 1 (JA), 9 (NL), 5 (PU), 8 (SL)	0/28
*P. melanotis*	75 (EM), 1 (MH), 59 (VZ)	3/135
*P. merriami*	6 (SO)	0/6
*P. mexicanus*	13 (CH), 11 (VZ)	0/24
*P. ochraventer*	11 (SL)	2/11
*P. pectoralis*	2 (CU), 1 (TM)	0/3
*P. schmidlyi*	1 (SO)	0/1
*P. spicilegus*	24 (JA), 7 (MH)	2/31
*P. truei*	1 (CI), 1 (TM)	0/2
*P. zarhynchus*	2 (CH)	0/2
*Peromyscus* spp.	1 (CI), 7 (EM), 8 (MH), 27 (NA)	1/43
*Reithrodontomys bakeri*	3 (GR)	0/3
*R. fulvescens*	1 (CI), 3 (JA), 4 (NL), 1 (OA), 1 (PU), 5 (SL), 1 (VZ)	0/16
*R. megalotis*	4 (EM), 4 (JA), 4 (NL), 5 (SL), 7 (TL), 1 (TM), 20 (VZ)	5/45
*R. mexicanus*	6 (JA), 14 (MH), 1 (SL)	0/21
*R. microdon*	3 (MH), 1 (OA)	1/4
*R. sumichrasti*	3 (CH), 18 (GR), 2 (MH)	5/23
*Reithrodontomys* spp.	1 (EM)	0/1
*Sigmodon alleni*	1 (JA)	0/1
*S. hispidus*	3 (TM)	0/3
*S. mascotensis*	11 (CH), 2 (GJ), 1 (GR), 11 (JA), 2 (MH), 14 (OA)	0/41
*S. ochrognathus*	1 (CI)	0/1
*S. toltecus*	1 (NL), 1 (TM), 18 (VZ)	0/20
Total		35/876

*CH, Chiapas (municipalities of Mapastepec, Ocozocoautla de Espinosa, and Zinacantán); CI, Chihuahua (Cusihuiriáchi); CU, Coahuila (Monclova); EM, Estado de México (Ecatepec de Morelos, Toluca, and Villa del Carbón); GJ, Guanajuato (Allende); GR, Guerrero (Chilpancingo de los Bravo); JA, Jalisco (Autlán de Navarro, Cocula, Jocotepec, and Ojuelos de Jalisco); MH, Michoacán (Múgica, Uruapan, Zinapécuaro, and Zitácuaro); NA, Nayarit (San Blas and Santa María del Oro); NL, Nuevo León (Doctor Arroyo, Galeana, Monterrey, and Santiago); OA, Oaxaca (Oaxaca de Juárez, San Pedro Mixtepec, and Santo Domingo Zanatepec); PU, Puebla (Tehuacán); SL, San Luis Potosí (Catorce and Ciudad del Maíz); SI, Sinaloa (Rosario); SO, Sonora (Navojoa and Yécora); TL, Tlaxcala (Tepetitla de Lardizabal); TM, Tamaulipas (San Fernando and Soto la Marina); VZ, Veracruz (Coatzacoalcos, Perote, and Poza Rica). †Number positive/number tested for antibody (IgG) against hantavirus.

**Table A2:** Prevalence of anti-hantavirus antibody (IgG) in rodents captured at 16 localities in 14 municipalities in 10 Mexican states*

Locality†	Bmus	Btay	Ocou	Pere	Phyl	Pleu	Plev	Pman	Pmeg	Pmel	Poch	Pspi	Pspp	Rmic	Rmeg	Rsum	Other‡	Total
1	–	–	1/7	–	–	–	–	–	–	–	–	–	–	–	–	–	–	1/7
2	1/6	–	–	–	–	–	–	–	–	–	–	–	–	–	–	–	0/4	1/10
3	0/1	–	–	–	–	–	0/2	–	1/29	–	–	–	–	–	–	3/18	0/15	4/65
4	–	1/13	–	–	–	–	–	–	–	–	–	2/9	–	–	–	–	0/18	3/40
5	–	–	–	–	2/8	–	0/1	–	–	2/9	–	–	–	–	–	–	–	4/18
6	–	–	–	–	–	–	–	–	–	0/1	–	–	0/1	1/1	–	2/2	0/16	3/21
7	–	–	–	–	–	–	–	–	–	–	–	–	1/27		–	–	0/3	1/30
8	–	–	–	1/5	–	0/1	–	1/9	–	–	–	–	–	–	0/3	–	0/25	2/43
9	–	–	–		–	–	3/21	–	–	–	–	–	–	–	0/1	–	–	3/22
10	–	–	–	0/6	–	–	–	1/5	–	–	–	–	–	–	0/5	–	0/26	1/42
11	–	–	–	–	–	–	1/16	–	–	–	2/11	–	–	–	–	–	0/4	3/31
12	–	–	–	–	–	1/7	–	–	–	–	–	–	–	–	–	–	0/4	1/11
13	–	0/1	–	–	–	1/6	–	–	–	–	–	–	–	–	–	–	0/3	1/10
14	–	–	–	–	–	–	–	0/4	–	0/2	–	–	–	–	3/8	–	–	3/14
15	0/1	–	–	–	–	–	–	1/1	–	0/23	–	–	–	–	1/9	–	0/6	2/40
16	–	–	–	–	–	–	–	–	–	1/34	–	–	–	–	1/3	–	0/3	2/40
Total	1/8	1/14	1/7	1/11	2/8	2/14	4/40	3/19	1/29	3/69	2/11	2/9	1/28	1/1	5/29	5/20	0/127	35/444

*Bmus, *Baiomys musculus*; Btay, *B. taylori*; Ocou, *Oryzomys couesi*; Pere, *Peromyscus eremicus*; Phyl, *P. hylocetes*; Pleu, *P. leucopus*; Plev, *P. levipes*; Pman, *P. maniculatus*; Pmeg, *P. megalops*; Pmel, *P. melanotis*; Poch, *P. ochraventer*; Pspi, *P. spicilegus*; Pspp, *Peromyscus* species; Rmeg, *Reithrodonomys megalotis*; Rmic, *R. microdon*; Rsum, *R. sumichrasti*; –, none collected or tested. †Locality 1: municipality of Mapastepec, state of Chiapas (latitude 15°25′10.861′′N, longitude 93°4′19.058′′W), 500 trap-nights (TNs) in July 2006; locality 2: Ocozocoautla de Espinosa, Chiapas (16°33′0.227′′N, 93°27′36.450′′W), 200 TN, July 2006; locality 3: Chilpancingo de los Bravo, Guerrero (17°39′5.203′′N, 99°50′12.010′′W), 450 TNs, July 2000; locality 4: Autlán de Navarro, Jalisco (19°49′53.997′′N, 104°26′43.206′′W), 500 TNs, July 2008; locality 5: Ecatepec de Morelos, México (19°48′10.195′′N, 99°39′37.575′′W), 400 TN, July 1998; locality 6: Uruapan, Michoacán (19°25′36.269′′N, 102°14′38.698′′W), 450 TNs, July 2008; locality 7: Santa María del Oro, Nayarit (21°39′35.991′′N, 104°25′15.079′′W), 500 TNs, July 2008; locality 8: Doctor Arroyo, Nuevo León (23°42′22.907′′N, 100°16′37.045′′W), 400 TN, August 2005; locality 9: Santiago, Nuevo León (25°22′7.222′′N, 100°5′12.090′′W), 450 TNs, July 2006; locality 10: Real de Catorce, San Luis Potosі (23°49′5.171′′N, 100°49′54.274′′W), 800 TNs, August 2005; locality 11: Ciudad del Maíz, San Luis Potosі (22°29′46.388′′N, 99°25′15.899′′W), 400 TNs, July 2006; locality 12: San Fernando, Tamaulipas (24°30′54.186′′N, 100°12′2.775′′W), 400 TNs, August 2005; locality 13: Soto la Marina, Tamaulipas (24°0′36.129′′N, 98°20′38.941′′W), 450 TNs, July 2008; locality 14: Perote, Veracruz (19°36′31.622′′N, 97°11′41.860′′W), 200 TNs, July 2006; locality 15: Perote, Veracruz (19°31′.748′′N, 97°9′23.002′′W), 200 TNs, July 2006; locality 16: Perote, Veracruz (19°31′38.073′′N, 97°9′.199′′W), 250 TN, July 2006. ‡Locality 2: *Oryzomys mexicanus* (n = 3), *Peromyscus mexicanus* (n = 1); locality 3: *Megadontomys thomasi* (n = 1), *Neotoma picta* (n = 2), *P. beatae* (n = 5), *P. evides* (n = 4), *Reithrodontomys bakeri* (n = 3); locality 4: *Osgoodomys banderanus* (n = 2), *R. mexicanus* (n = 5), *Sigmodon alleni* (n = 1), *S. mascotensis* (n = 10); locality 6 : *Neotoma mexicana* (n = 1), *Neotomodon alstoni* (n = 1), *R. mexicanus* (n = 14); locality 7: *N. mexicana* (n = 3); locality 8: *Neotoma leucodon* (n = 10), *Onychomys leucogaster* (n = 1), *P. melanophrys* (n = 9), *R. fulvescens* (n = 4), *S. toltecus* (n = 1); locality 10: *N. leucodon* (n = 10), *O. leucogaster* (n = 3), *P. melanophrys* (n = 8), *R. fulvescens* (n = 5); locality 11: *Oryzomys melanotis* (n = 4); locality 12: *Neotoma micropus* (n = 2), *O. leucogaster* (n = 1), *S. toltecus* (n = 1); locality 13: *P. pectoralis* (n = 1), *S. hispidus* (n = 2); locality 15: *Habromys* sp. (n = 1), *P. beatae* (n = 4), *R. fulvescens* (n = 1); locality 16: *N. mexicana* (n = 2), *P. mexicanus* (n = 1).

**Table A3:** Nonidentities among the amino acid sequences of a 359-aa fragment of the G_C_ glycoproteins of 14 hantaviruses from Mexico and 5 other hantaviruses from North America

Virus	Strain	Virus or strain																		
		M0040008	H0460041	M0040059	H0460023	B0030008	H0460008	M0040049	H0030065	H0020015	H0460038	H0030073	CARV	RM-97	HUIV	LSCV	MTNV	NYV	SNVCC74	SNVBR
ELMCV	M0040008	–	7.8	7.5	16.4	16.3	16.7	15.6	15.9	15.9	15.3	15.6	0.0	9.5	7.5	15.9	17.3	16.2	15.6	15.6
ELMCV	H0460041		–	1.1	15.3	16.1	15.3	17.3	16.4	16.4	15.6	15.9	7.8	7.5	1.9	15.6	18.1	16.4	15.3	16.4
ELMCV	M0040059			–	15.3	16.1	15.3	17.3	16.4	16.4	15.6	15.9	7.5	7.2	2.2	15.6	18.4	16.4	15.3	16.4
LSCV	H0460023				–	2.8	0.8	4.5	13.9	14.2	14.8	15.3	16.4	17.0	14.2	2.2	6.4	14.8	13.9	15.6
LSCV	B0030008					–	2.5	5.4	14.9	15.2	14.6	14.9	16.3	16.9	14.9	1.7	6.5	14.4	13.5	15.2
LSCV	H0460008						–	5.3	14.5	14.8	14.8	15.3	16.7	17.3	14.2	2.2	6.4	14.8	13.9	15.6
LSCV	M0040049							–	14.5	14.8	15.3	15.6	15.6	17.0	16.2	5.0	7.0	15.0	14.8	15.9
SNV	H0030065								–	0.3	2.2	3.9	15.9	18.1	15.6	14.5	14.8	3.9	2.5	3.9
SNV	H0020015									–	2.5	4.2	15.9	18.1	15.6	14.8	15.0	4.2	2.8	4.2
SNV	H0460038										–	4.2	15.3	17.5	14.8	14.2	15.0	4.2	3.3	4.7
SNV	H0030073											–	15.6	18.4	15.3	14.2	15.3	3.6	3.1	1.1
CARV	26/2006												–	9.5	7.5	15.9	17.3	16.2	15.6	15.6
ELMCV	RM-97													–	7.2	16.4	18.4	18.9	17.3	17.8
HUIV	200/2006														–	14.5	17.0	15.6	14.5	15.9
LSCV	LSCV															–	6.1	13.6	12.8	14.5
MTNV	104/2006																–	14.8	13.9	15.6
NYV	RI-1																	–	1.9	4.2
SNV	CC74																		–-	3.1
SNV	BR																			–

*CARV, Carrizal virus; ELMCV, El Moro Canyon virus; HUIV, Huitzilac virus; LSCV, Limestone Canyon virus; MTNV, Montano virus; NYV, New York virus; SNV, Sin Nombre virus ; CC74, SNV Convict Creek strain74; BR, SNV Blue River-Oklahoma strain; –, diagonal line; underline, lowest amino acid sequence nonidentify (or closest genetic identity) with known viruses.

**Table:** Hantaviruses found in 24 of 35 antibody-positive rodents captured in Mexico in 1998–2008, by state*†

Virus	Strain	Rodent						GenBank accession no.	
		Museum no.	Species	State	Date captured	Antibody titer		S segment	M segment
ELMCV	M0040008	TK93357	*Reithrodontomys sumichrasti*	GR (12)	2000 Jul 20	>5,120		JN097454	JN097478
ELMCV	M0040010	TK93368	*R. sumichrasti*	GR (12)	2000 Jul 20	>5,120		JN097455	ND
ELMCV	M0040012	TK93370	*R. sumichrasti*	GR (12)	2000 Jul 20	>5,120		JN097456	ND
LSCV	M0040047	TK148818	*Peromyscus spicilegus*	JA (8)	2008 Jul 22	>5,120		JN097457	ND
LSCV	M0040049	TK148820	*P. spicilegus*	JA (8)	2008 Jul 22	>5,120		JN097458	JN097479
LSCV	B0030007	TK78282	*P. melanotis*	EM (10)	1998 Jun 24	>5,120		JN097459	ND
LSCV	B0030008	TK78283	*P. hylocetes*	EM (10)	1998 Jun 24	>5,120		JN097460	JN097480
LSCV	B0030015	TK78290	*P. hylocetes*	EM (10)	1998 Jun 24	>5,120		JN097461	ND
SNV	H0030065	TK137297	*P. maniculatus*	NL (4)	2005 Aug 6	>5,120		JN097462	JN097481
SNV	H0030067	TK137312	*P. eremicus*	NL (4)	2005 Aug 6	>5,120		JN097463	ND
LSCV	H0460002	TK150003	*P. levipes*	NL (1)	2006 Jul 12	>5,120		JN097464	ND
LSCV	H0460005	TK150006	*P. levipes*	NL (1)	2006 Jul 12	>5,120		JN097465	ND
LSCV	H0460008	TK150017	*P. levipes*	NL (1)	2006 Jul 12	>5,120		JN097466	JN097482
SNV	H0020015	TK133396	*P. maniculatus*	SL (3)	2005 Aug 4	>5,120		JN097467	JN097483
LSCV	H0460017	TK150043	*P. ochraventer*	SL (7)	2006 Jul 13	>5,120		JN097468	ND
LSCV	H0460023	TK150086	*P. ochraventer*	SL (7)	2006 Jul 13	>5,120		JN097469	JN097484
SNV	H0030073	TK137359	*P. leucopus*	TM (2)	2005 Aug 8	>5,120		JN097470	JN097485
ELMCV	M0040059	TK150090	*R. megalotis*	VZ (11)	2006 Jul 15	320		JN097471	JN097486
ELMCV	H0460026	TK150101	*R. megalotis*	VZ (11)	2006 Jul 15	>5,120		JN097473	ND
ELMCV	H0460029	TK150117	*P. melanotis*	VZ (11)	2006 Jul 16	1,280		JN097472	ND
ELMCV	H0460032	TK150161	*R. megalotis*	VZ (11)	2006 Jul 15	320		JN097474	ND
ELMCV	H0460035	TK150163	*R. megalotis*	VZ (11)	2006 Jul 16	320		JN097475	ND
SNV	H0460038	TK150166	*P. maniculatus*	VZ (11)	2006 Jul 16	>5,120		JN097476	JN097487
ELMCV	H0460041	TK150182	*R. megalotis*	VZ (11)	2006 Jul 16	1,280		JN097477	JN097488

*Numbers in parentheses indicate locations on the map in Figure 1. S, small; M, medium; ELMCV, El Moro Canyon virus; GR, Guerrero; ND, sequences not determined; LSCV, Limestone Canyon virus; JA, Jalisco; EM, México (state); SNV, Sin Nombre virus; NL, Nuevo León; SL, San Luis Potosí; TM, Tamaulipas; VZ, Veracruz. †Antibody-positive, hantavirus RNA–negative rodents (species, location, antibody titer): TK78287 (*P. melanotis*, EM, 1,280); TK93383 (*P. megalops*, GR, >5,120); TK148439 (*Peromyscus* sp., Nayarit, 1,280); TK148793 (*Baiomys taylori*, JA, 320); TK148836 (*R. microdon*, Michoacán, 1,280); TK148842 (*R. sumichrasti*, Michoacán, 320); TK148845 (*R. sumichrasti*, Michoacán, 1,280); TK148984 (*P. leucopus*, Tamaulipas, >5,120); TK150045 (*P. levipes*, SL, >5,120); TK150515 (*B. musculus,* Chiapas, 320); and TK150518 (*Oryzomys couesi*, Chiapas, 1,280).
